# Reliability of the adapted compensatory arm and leg movements scale during perturbation treadmill walking in older adults

**DOI:** 10.3389/fspor.2025.1658856

**Published:** 2025-09-17

**Authors:** Michael Schwenk, Mitzi Ramirez Mantilla, Nina Marie Schmidt, Vanessa Haug, Christian Werner, Markus Gruber, Michael Denkinger, Tim Fleiner

**Affiliations:** ^1^ Department of Sport Science, Human Performance Research Centre, University of Konstanz, Konstanz, Germany; ^2^ Department for Health Services Research, Geriatric Medicine, School of Medicine and Health, Carl von Ossietzky University, Oldenburg, Germany; ^3^ Institute for Geriatric Research, Ulm University, Ulm, Germany; ^4^ Geriatric Center, Agaplesion Bethesda Clinic, Ulm, Germany; ^5^ Geriatric Center, Medical Faculty Heidelberg, Heidelberg University, Heidelberg, Germany; ^6^ Institute of Medical Engineering and Mechatronics, Ulm University of Applied Sciences, Ulm, Germany

**Keywords:** reactive balance, assessment, perturbation, treadmill, walking, reliability

## Abstract

**Introduction:**

Reactive balance during walking is crucial for fall prevention, as it determines recovery from unexpected perturbations like slips and trips. Existing reactive balance assessments are complex and lab-based, lacking an easy-to-use alternative for broader application in clinical environments. The Adapted Compensatory Arm and Leg Movements (A-CALM) scale was developed to address this gap by providing an observer-based tool to evaluate compensatory balance reactions during perturbation treadmill walking. This study assessed its inter- and intra-rater reliability in fall-prone older adults.

**Methods:**

Eighteen participants aged 82 ± 7 years walked on the BalanceTutor® perturbation treadmill. Depending on assigned intensity levels, each received 8, 16, or 24 perturbations in mediolateral and anteroposterior directions. Compensatory balance reactions after each perturbation were video-recorded and evaluated by three trained raters using the A-CALM scale, capturing responses from minor adjustments to near-fall scenarios. Arm movements were rated on a five-point scale (1 = near fall, 5 = regular arm swing), while leg movements were rated on an eight-point scale (1 = near fall, 8 = normal walking), with intermediate scores reflecting varying recovery steps. Inter-rater reliability was assessed using Fleiss' Kappa, while intra-rater reliability over a two-week interval was evaluated using Cohen's Kappa.

**Results:**

Overall, 288 perturbations were recorded. The A-CALM scale demonstrated strong intra-rater reliability, with Kappa values of 0.85 (95% CI = 0.80–0.89) for arm scores, 0.80 (95% CI = 0.75–0.86) for leg scores, and 0.86 (95% CI = 0.83–0.90) for total scores, indicating a high level of consistency in the raters' assessments across time. Inter-rater reliability was substantial for arm scores (*K* = 0.67, 95% CI = 0.62–0.72) but moderate for leg scores (*K* = 0.48, 95% CI = 0.44–0.51) and total scores (*K* = 0.41, 95% CI = 0.38–0.44) with significant values in all analyses (*p* < 0.001).

**Conclusion:**

The A-CALM scale showed high intra-rater consistency and moderate-to-substantial inter-rater agreement, with greater reliability for arm than leg movements. Single-rater use is recommended to enhance stability, while future work should refine leg scoring and validate the scale in larger cohorts with outcomes like falls and functional decline.

## 1 Introduction

Falls are a leading cause of injury, loss of independence, and reduced quality of life, particularly in older adults and individuals with balance impairments (1). Many falls occur during walking, often triggered by trips or other unexpected perturbations that challenge reactive balance (2). Reactive balance—the ability to regain stability after an external disturbance—is a critical predictor of fall risk (3). However, its assessment is typically limited to laboratory settings using specialized equipment such as motion capture systems and force plates (4–6). While these approaches provide precise data, they require extensive resources, making them impractical for routine clinical practice. The lack of a clinically feasible method for assessing reactive balance during walking creates a significant gap in fall risk evaluation and prevention. With the rising demand for effective fall risk screening in hospitals, rehabilitation centers, and long-term care facilities, there is a clear need for a practical, observer-based tool that can reliably assess reactive balance in clinical settings.

Established tools such as the Dynamic Gait Index (DGI) (7) and Romberg Test (8) are valuable for evaluating general balance and mobility but do not adequately capture reactive balance. The Romberg Test measures static balance under eyes-open or eyes-closed conditions and does not expose individuals to external disturbances. The DGI, while focused on gait, emphasizes voluntary, anticipatory tasks (e.g., walking with head turns or over obstacles) that rely on feedforward control. Neither tool assesses how individuals respond in real time to sudden, high-magnitude, and unpredictable perturbations—the very situations in which falls most often occur.

Observer-based assessments of reactive balance have primarily focused on stance perturbations, such as the Stepping Threshold Test (9) and the Compensatory Arm and Leg Movements (CALM) scale (10). These tools allow clinicians to evaluate postural responses to external disturbances without specialized laboratory equipment. To date, only one study has developed a rating method for treadmill-induced perturbation (11). However, the scale is limited to assessing the extent of support and the quality of stepping responses. Its validity was only moderate, reliability was not examined, and importantly, it does not account for upper extremity movements, even though arm reactions are a key compensatory strategy after perturbations (12).

To our knowledge, no observer-based tool currently evaluates both upper- and lower-extremities compensatory movements during walking. In this regard, the CALM scale is particularly promising, as it incorporates both arms and legs—critical components of effective balance recovery (10). Originally developed to assess responses to large, mediolateral, unpredictable stance perturbations, the CALM scale uses a structured scoring system based on movement amplitude and stability. Arm movements are rated from 1 (grasping/hanging, least stable) to 5 (motionless, most stable), while leg movements are scored from 2 (multiple steps) to 9 (motionless, most stable), covering strategies such as stepping, swinging, and sliding. Although compensatory actions like stepping or widening the base are effective for preventing falls, the CALM scale prioritizes stability, with higher scores assigned to responses requiring minimal movement. The CALM scale has demonstrated excellent intra-rater reliability (*K* > 0.97) and moderate-to-high inter-rater reliability (*K* = 0.46–0.88) (10). Its validity was supported by significant negative correlations (*r* = −0.48 to −0.81) between CALM scores and kinematic measurements of movement amplitude obtained with a Vicon motion capture system, confirming its ability to capture key aspects of reactive postural control (10).

However, the CALM scale was originally developed to evaluate responses to perturbations in standing and has not yet been applied to walking. Adapting the scale to walking perturbations could provide a clinically feasible assessment tool, enabling routine screening of individuals at risk of falls in real-world settings. Such an approach may support early intervention, guided targeted rehabilitation, and facilitate large-scale fall risk assessment without the need for expensive and time-consuming laboratory equipment.

The present study aimed to 1. adapt the CALM scale for assessing reactive balance responses to walking perturbations on a perturbation treadmill and 2. determine the reliability of the adapted version (A-CALM), ensuring its consistency as a clinical tool. We hypothesized that the A-CALM scale would demonstrate reliability outcomes comparable to that of the original CALM scale.

## 2 Materials and methods

### 2.1 Adaptation of the CALM scale

The CALM scale (10), originally designed for stance perturbations, was adapted for perturbation treadmill walking through expert discussions (MS, MRM, NMS, VH, and TF). This process involved analyzing key differences between static and dynamic balance recovery. In particular, experts considered factors such as the number of recovery steps during perturbed walking and the role of arm movements in locomotion. To create the A-CALM scale, the descriptions of the original CALM items were refined to reflect stabilization strategies unique to walking perturbations.

The adaptation followed a structured process:

1. Review of original items: Each CALM item was examined for its applicability to dynamic treadmill walking.
2. Identification of modification needs: For each item, we determined whether differences in baseline movement (e.g., natural arm swing in walking) or in perturbation demands (e.g., multiple recovery steps) required changes in thresholds or descriptions.
3. Consensus: All changes were discussed until unanimous agreement was reached. A modification was accepted only if all five experts agreed that it improved face validity for walking perturbations.

The results of the adaptation process are present in the results section.

#### 2.1.1 Perturbation treadmill protocol

The perturbation treadmill protocol consisted of three blocks with increasing levels of difficulty (Table 1). Each block included eight perturbations that randomly occurred either in the forward (treadmill slows down), backward (treadmill accelerates), or sideways (treadmill moves sideways) direction. Two types of perturbations were tested: mediolateral (four to the left and right) and anteroposterior (four forward and backward). Both types were applied at each difficulty level (see Supplementary Materials, Table S5).

**Table 1:** Perturbation-treadmill blocks and corresponding intensity levels and numbers of perturbations.

Perturbation-treadmill block	Perturbation level	Intensity of perturbations^a^	Number and type of perturbations
Block 1	Level 1	ML: 10, AP: 15	4 ML (left/right) 4 AP (forward/backwards) Total 8
Block 2	Level 2	ML: 15, AP: 20	4 ML (left/right) 4 AP (forward/backwards) Total 8
Block 3	Level 3	ML: 20, AP: 25	4 ML (left/right) 4 AP (forward/backwards) Total 8

ML, mediolateral. AP, anteroposterior.

^a^ Perturbation treadmill (BalanceTutor®) provides 30 intensity levels (1–30), with the corresponding accelerations and displacements detailed in the Supplementary Material, Table S5.

We assumed that the mediolateral (ML) perturbations were more challenging than the anteroposterior (AP) perturbations. This assumption was based on literature showing that ML perturbations are significantly more difficult to compensate for than AP perturbations and therefore require more compensatory protective steps (13).

The frequency of perturbations varied randomly between 8 and 12 s. Difficulty increased across blocks by raising perturbation intensity (Table 1). Walking speed was determined using the 4-m walk test from the SPPB. Participants first walked at 50% of this speed, then the speed was gradually increased until they said “stop,” establishing their preferred walking speed (14).

All participants began at difficulty level 1. Progression to levels 2 and 3 depended on step responses. During perturbations, single steps received 1 point, while multiple steps received no points. Single steps were defined as one recovery step; multiple steps were defined as two or more deviations from the normal gait pattern. Each block consisted of 8 perturbations, and participants were instructed to proceed to the next level if they achieved at least 4 points within a level (e.g., 4 or more single steps). This design ensured that each participant experienced perturbations of sufficient intensity to elicit clear balance reactions.

Each block was video-recorded for later A-CALM analysis. Participants were instructed to maintain stability, take as few compensatory steps as possible, and remain within their support base.

#### 2.1.2 Video-based completion of the A-CALM scale

Video recordings of all perturbations were assessed by three trained raters (MRM, NMS, VH) using the A-CALM scale. Training involved familiarization with the scale, calibration sessions with example videos, and group discussions to resolve discrepancies, supported by expert feedback (MS and TF) to ensure standardization. Raters typically achieved stable proficiency after approximately 4–6 h of structured training over 4–5 sessions (see Supplementary Materials, Description S1). The training emphasized consistent application of the scoring system: arm score (range 1–5, Table 2), leg score (range 1–8, Table 3), and total score (range 2–13).

**Table 2:** Compensatory arm movement module of the original CALM scale (10) and the adapted CALM scale.

Compensatory arm movements				
Original CALM (10)		Adapted CALM		Justification
Item (Score)	Item description	Item (Score)	Item description	
1	**Near fall.** Being supported by the safety device or grasping a near surface.	1	**Near fall.** Being supported by the safety device or grasping *one or both safety ropes.*	It was a specific safety rope used in our protocol to reflect the treadmill environment.
2	**Large amplitude.** Large shoulder abduction raising the hand(s) above the shoulders height.	2	**Large amplitude.** Large shoulder abduction raising the *elbow(s)* above the shoulders height.	Abduction measurements from the elbow provided a more accurate and practical approach.
3	**Moderate amplitude.** Moderate shoulder abduction raising the hand(s) below the shoulders height.	3	**Moderate amplitude**. Moderate shoulder abduction raising the *elbow(s)* below the shoulders height.	Abduction measurements from the elbow provided a more accurate and practical approach.
4	**Small amplitude.** Minor shoulder abduction, with contact loss between the hand(s) and the leg(s) up to about 10 cm.	4	**Small amplitude**. Minor shoulder abduction. *Angle between shoulder(s) and elbow(s) greater than 45 degrees. But arm movement(s) are visible, which go(es) beyond the normal arm swing when walking.*	Angle-based measurements provided a more accurate and practical approach.
5	**Motionless.** Keeping the hands in contact with the legs while recovering body equilibrium.	5	***Regular arm swing****. Maintaining the regular arm swing* while recovering body equilibrium.	The focus was maintaining each participant's individual normal walking pattern.

Adaptations are highlighted in *italics*.

**Table 3:** Compensatory leg movement module of the original CALM scale (10) and the adapted CALM scale.

Compensatory arm movements				
Original CALM (10)		Adapted CALM		Justification
Item) (Score)	Item description	Item (Score)	Item description	
1	**Near fall.** Being supported by the safety device or grasping a near surface, regardless of leg movements.	1	**Near fall.** Being supported by the safety device or *grasping one or both safety ropes,* regardless of leg movements.	It was a specific safety setup used in our protocol to reflect the treadmill environment.
2	**Multiple steps through large increment of the support base.** Changing the support base for balance recovery through two or more steps, regardless the stepping pattern, with total displacement larger than 15 cm.	2	**Extensive recovery stepping**^a^ **through large increment of the support base.** Balance recovery through *three or more recovery steps*^a^*, with at least one foot (partly) outside a 40 cm corridor marked on the treadmill.*	Older adults with balance impairments often require over three steps to recover. We replaced the displacement measure with support base increment, as the base is already ∼10 cm during regular walking (27).
3	**Multiple steps through small increment of the support base.** Changing the support base for balance recovery through two or more steps, regardless the stepping pattern, with total displacement equal to or smaller than 15 cm.	3	***Extensive recovery stepping* through small increment of the support base**. Balance recovery through *three or more recovery steps*^a^*, with both feet within a 40 cm corridor marked on the treadmill.*	We used the same justification as for item 2.
4	**Single step through large increment of the support base.** Balance recovery through a single step, regardless the stepping pattern, with stepping amplitude larger than 15 cm.	4	***Minor recovery stepping***^a^ **through large increment of the support base.** *Balance recovery by two or less recovery steps*^a^*, with at least one foot (partly) outside a 40 cm corridor marked on the treadmill.*	We used the same justification as for item 2.
5	**Single step through small increment of the support base.** Balance recovery through a single step, regardless the stepping pattern, with stepping amplitude equal to or smaller than 15 cm.	5	***Minor recovery stepping***^a^ **through small increment of the support base.** *Balance recovery by two or less recovery steps*^a^*, with both feet within a 40 cm corridor marked on the treadmill.*	We used the same justification as for item 2.
6	**Leg swing with large amplitude**. Swinging one leg outward for counter-weighting lateral body leaning while supporting the whole body on the other leg, with movement amplitude larger than 15 cm.	6	**Leg swing with large amplitude**. Swinging one leg outward for counter-weighting lateral body leaning while supporting the whole body on the other leg, with *leg abduction larger than 20 degrees.*	Angle-based measurements provided a more accurate and practical approach.
7	**Leg swing with small amplitude**. Swinging one leg outward for counter-weighting trunk movements while supporting the whole body on the other leg, with movement amplitude equal to or smaller than 15 cm.	7	**Leg swing with small amplitude**. Swinging one leg outward for counter-weighting trunk movements while supporting the whole body on the other leg, with *leg abduction less than 20 degrees.*	Angle-based measurements provided a more accurate and practical approach.
8	**Sliding.** Short one-foot or two-feet outward sliding over the support base (no feet-ground contact loss), not crossing the 5 cm mark on the platform, or short (few centimeters) one-foot rising above the ground landing at about the place.		(Item deleted)	Item was removed as the sliding pattern was not observed in our treadmill-based perturbations.
9	**Motionless.** Balance recovery keeping the feet in place.	8	***Walking*** *. Normal walking pattern on treadmill.*	The focus was maintaining each participant's individual normal walking pattern.

Adaptations are highlighted in *italics*.

^a^ Recovery step is defined as any deviation from normal walking that involves an adjustment in foot placement to preserve balance.

The reliability assessments were conducted by three raters with complementary expertise: MRM as a sport scientist, with a background in rehabilitation and public health; VH, a sport scientist specializing in geriatric care and fall prevention; and NMS, a sport therapist with experience in fall risk assessment (Full rater details are provided in Supplementary Materials, Description S1).

All assessments were video-based; no real-time observations were conducted. This ensured standardized scoring across participants and allowed raters to replay movements if needed. Recordings came from a single camera angle, chosen to balance consistency and quality with space limitations. Although additional angles could have offered more detail, this setup provided reliable data within the practical constraints.

For inter-rater reliability, each rater independently scored all videos and was blinded to others' evaluations. Scores were submitted to the study management and compared across raters. Intra-rater reliability was assessed by comparing the consistency of MRM's ratings across a two-week interval.

Raters followed a predefined rating schedule. Based on the typical video length, we estimate that scoring one participant for a single level takes approximately 10–15 min for an experienced rater. This estimate assumes an average of ∼2 min per perturbation to a. view the perturbation video clip, b. pause/replay briefly if needed to confirm arm and leg items, and c. record arm, leg and total scores (see Supplementary Materials, Description S1).

### 2.2 Study participants

Participants were recruited from the AGAPLESION BETHESDA KLINIK ULM, including patients from the acute clinic, inpatient and outpatient rehabilitation, and the outpatient geriatric clinic. Inclusion criteria were: age ≥65 years, Charité Mobility Index (CHARMI®) score of 6 or higher (11), and the ability to be mobile without a walking aid.

Exclusion criteria were: a. palliative care, b. inability to provide informed consent, c. limited communication ability, d. inability to walk on a treadmill at habitual walking speed for 2 min, e. uncontrolled cardiovascular, metabolic, or psychiatric conditions, f. presence of a permanent infusion or continuous oxygen device use, g. body weight >135 kg, h. severe osteoporosis, i. current fractures, j. open or chronic wounds, and k. surgery within the past eight weeks.

#### 2.2.1 Ethical considerations

The study was conducted following ethical guidelines and was approved by the ethical commission at the University Hospital Ulm (reference number 73/24). Informed consent was obtained from all participants, ensuring they were fully aware of the study procedures and any associated risks.

### 2.3 Descriptive measures

Established validated assessments were used to characterize the functional status and fall risk of the study participants. The Charité Mobility Index (CHARMI®) score is a comprehensive tool that assesses mobility impairment in elderly individuals by considering gait, balance, and functional mobility (15).

The Clinical Frailty Scale (CFS) categorizes patients based on their level of frailty, ranging from very fit to severely frail (16).

The Short Physical Performance Battery (SPPB) evaluates lower extremity function in older adults using balance tests, a gait speed test, and a chair stand test (17). Each component is scored from 0 to 4. The total score ranges from 0 to 12, with higher scores reflecting better mobility.

The Timed Up and Go (TUG) test assesses mobility and fall risk by timing how long a person takes to stand up from a chair, walk three meters, turn, and return. A longer completion time indicates reduced mobility and a higher risk of falls (18).

The Short Falls Efficacy Scale-International (Short FES-I) measures fear of falling in older adults through seven questions about daily activities (19). Each item is scored from 1 (not at all concerned) to 4 (very concerned). Higher scores indicate greater fear of falling and potential activity avoidance.

### 2.4 Materials and equipment

#### 2.4.1 Perturbation treadmill

The BalanceTutor® (MediTouch Ltd., Israel) is an advanced treadmill system designed for perturbation-based balance assessment and training. It delivers controlled, reproducible perturbations in multiple directions to simulate real-world balance challenges. The treadmill induced reactive balance responses, which raters categorized using the A-CALM scale. Participants were secured with a safety harness to prevent falls. Perturbations were not provided manually but followed a standardized protocol and were triggered automatically. The built-in Center of Pressure (COP) was used to induce perturbations during the stance phase.

#### 2.4.2 Video recordings

**Figure 1:**
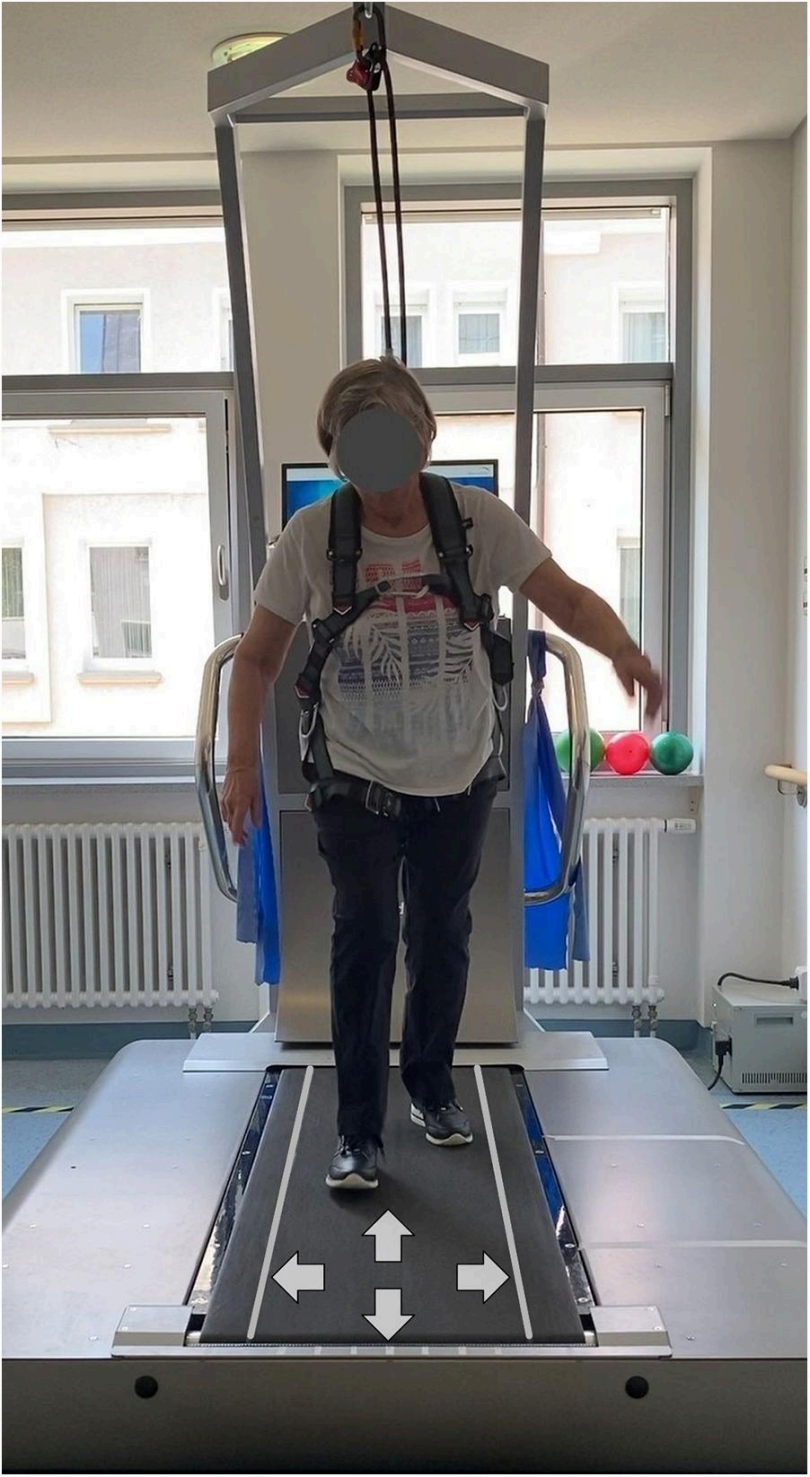
Frontal view of a participant walking on the BalanceTutor® perturbation treadmill (MediTouch Ltd., Israel) during the Adapted CALM assessment. Image captured by the study team.

Video data was collected with a smartphone camera (Apple iPhone SE, 3rd Generation) mounted on a tripod for stability and to ensure a consistent frontal view of the participants (Figure 1). The recordings served as a visual reference for raters to assess compensatory movements using the A-CALM scale. For the video-observation analysis, angles were measured with Kinovea (version 0.9.5), a free software for motion analysis.

### 2.5 Statistical analysis

All statistical analyses were conducted in R Statistical Software (v4.3.0; R Core Team 2021).

Inter-rater reliability: Agreement among the three raters was evaluated with Fleiss' Kappa, applied to the A-CALM arm score, leg score, and total score (20). To examine agreement between individual pairs of raters (Rater 1 vs. Rater 2, Rater 2 vs. Rater 3, Rater 1 vs. Rater 3), we used Cohen's Kappa (21). Kappa values were interpreted as follows: <0.00 poor, 0.00–0.20 slight, 0.21–0.40 fair, 0.41–0.60 moderate, 0.61–0.80 substantial, and >0.80 almost perfect agreement (22). A subanalysis examined whether agreement differed by perturbation direction, with Cohen's Kappa calculated separately for AP and ML perturbations.

Intra-rater reliability: Cohen's Kappa was also applied to determine the agreement of repeated ratings by the same rater across a 2-week interval. In addition, the reliability of cumulated A-CALM scores was assessed by perturbation level (Levels 1, 2, and 3). Cumulated scores were obtained by summing the total A-CALM scores from the eight perturbations, yielding a possible score range from 16 to 104. Intra-rater reliability of the cumulated scores was evaluated using intraclass correlation coefficients (ICC), model (3,1). This model emphasizes consistency by testing whether relative scoring patterns remain stable over time, even if a rater applies a small systematic bias (e.g., consistently scoring higher at the second session).

Inter-rater reliability (ICC): To test whether raters produced interchangeable scores, we calculated ICC model (2,1). Unlike the consistency model, this approach assesses whether raters give exactly the same values, making even small systematic differences relevant. ICC values were interpreted using standard thresholds: <0.50 indicate poor, 0.50–0.75 moderate, 0.75–0.90 good, and >0.90 excellent reliability (23).

Additional analyses: To test efficiency, we compared cumulative total scores derived from four perturbations (two AP and two ML) with those from the full eight perturbations (four AP and four ML). Intra-rater reliability for this comparison was examined with ICC(3,1), based on a two-way mixed-effects, single-rater model.

Sample size: Our design followed the original CALM study that included 12 participants × 18 trials, resulting in a total of 216 paired ratings (10). That study reported high intra-rater and moderate–to–very high inter-rater reliability. To increase precision, we expanded to 16 participants  × 18 trials, providing 288 paired ratings. With this sample, the study had >99% power to detect the expected reliability.

## 3 Results

### 3.1 Adaptation of the CALM scale

The adaptations made to create the A-CALM scale are summarized in Tables 2, 3. The basic structure of the original CALM scale (10), with its two modules—“Compensatory Arm Movements” and “Compensatory Leg Movements”—was maintained, as was the scoring system. Similar to the original CALM, participants' responses to treadmill perturbations are filmed in the frontal plane using a commercial digital camera for offline analysis based on the A-CALM scale. The main adaptations involved the description of individual items to better capture balance recovery during walking.

Raters first defined each participant's regular walking pattern, including natural arm and leg swing in the absence of perturbations. Slight movements were accepted as long as they did not represent a reactive response to perturbations. This individualized reference point was then used to evaluate deviations during balance recovery.

For the “Compensatory Arm Movements” module, the amplitude for item 4 (small amplitude) was increased compared to the original CALM (Table 2). This adjustment was made because natural compensatory arm movements occur even during unperturbed walking (24). In contrast, the original CALM assumed both arms rested beside the trunk with the palms lightly touching the thighs. In addition, item 5 was modified from standing “motionless” (original CALM) to “regular arm swing” during walking.

For the “Compensatory Leg Movements” module, the thresholds for the number of recovery steps were increased (items 2–5) were increased to account for treadmill perturbations (Table 3). Older adults with balance impairments often require more than three steps to regain stability after treadmill perturbations due to slower neuromuscular responses (25, 26). Accordingly, items 2 and 3 were revised to require three or more steps (instead of two or more in the original CALM). For items 4 and 5, the threshold was increased to two or fewer steps (instead of a single step). A recovery step was defined as any deviation from normal walking that involves an adjustment in foot placement to preserve balance (Table 3).

For items 6–7 the measurement unit was changed from centimeters to degrees, as angle-based measurements provided a more accurate and practical approach for our analysis. In cases where arm or leg abduction was ambiguous, Kinovea was used to determine the precise joint angles by marking anatomical reference points and calculating the movement relative to the body's neutral position. This approach improved the precision and consistency of the arm and leg item measurements across raters.

The thresholds for spatial parameters of recovery steps (i.e., increment of the support base) were increased in the A-CALM. A large increment was defined as at least one foot was partly or fully outside of a 40 cm corridor marked on the treadmill (Figure 1). This adjustment was necessary because the support base during normal walking is already about 10 cm during regular walking (27), whereas in the original CALM it was 0 cm, as participants stood in a Romberg position with their feet together.

The original CALM item “sliding” (item 8) was removed, as it was not relevant for walking perturbations. In addition, item 9 was modified from standing “motionless” to “walking” with a normal gait pattern on the treadmill.

### 3.2 Reliability study

#### 3.2.1 Participant's characteristics

The study included 18 participants (mean age: 82 ± 7 years, body height 169.2 ± 9.1 cm and body weight 69.8 ± 10.5 kg) with an even gender distribution (Table 4). Mobility and frailty assessments revealed no significant impairments (median CHARMI®: 10, CFS: 2). The median FES-I score of 8 indicated a low concern about falling. A median SPPB score of 8 reflected moderate physical performance, while a median TUG time of 11.0 s indicated mild mobility impairments. Overall, the sample was mildly physically impaired, with subtle mobility limitations that may increase fall risk in challenging situations.

**Table 4:** Participants' descriptive characteristics.

Characteristics	Participants (*n* = 18)
Age, years, mean ± SD (range)	81.5 ± 7.4 (69–92)
Body height, mean ± SD (range)	169.2 ± 9.1 (148–185)
Body weight, mean ± SD (range)	69.8 ± 10.5 (45–85)
Female, *n* (%)	9 (50)
CHARMI® score, median, (IQR)	10 (1)
CFS score, median, (IQR)	2 (3)
Short FES-I score, median, (IQR)	8 (3)
SPPB score, median, (IQR)	8 (4.25)
TUG score, median, (IQR)	10.97 (7.39)

CHARMI®, Charité Mobility Index; CFS, Clinical Frailty Scale; FES-I, Short Falls Efficacy Scale-International; SPPB, Short Physical Performance Battery; TUG, Timed Up and Go test; SD, standard deviation; IQR, interquartile range.

Further analysis of the SPPB scores showed that five participants had poor to severe functional limitations (scores 0–6), eight had moderate limitations (scores 7–9), and five had good to minimal limitations (scores 10–12). When grouped into “impaired” (scores 0–9) and “no significant balance issues” (scores 10–12), most participants fell into the impaired category. This distribution may have influenced how many were able to complete all levels of perturbation difficulty.

#### 3.2.2 Frequencies of A-CALM total scores

All participants (*n* = 18) completed the perturbation treadmill protocol at difficulty level 1. Twelve participants (67%) progressed to level 2, and six (33%) reached the highest difficulty level, 3. In total, 288 perturbations were recorded and analyzed by the three raters.

**Figure 2:**
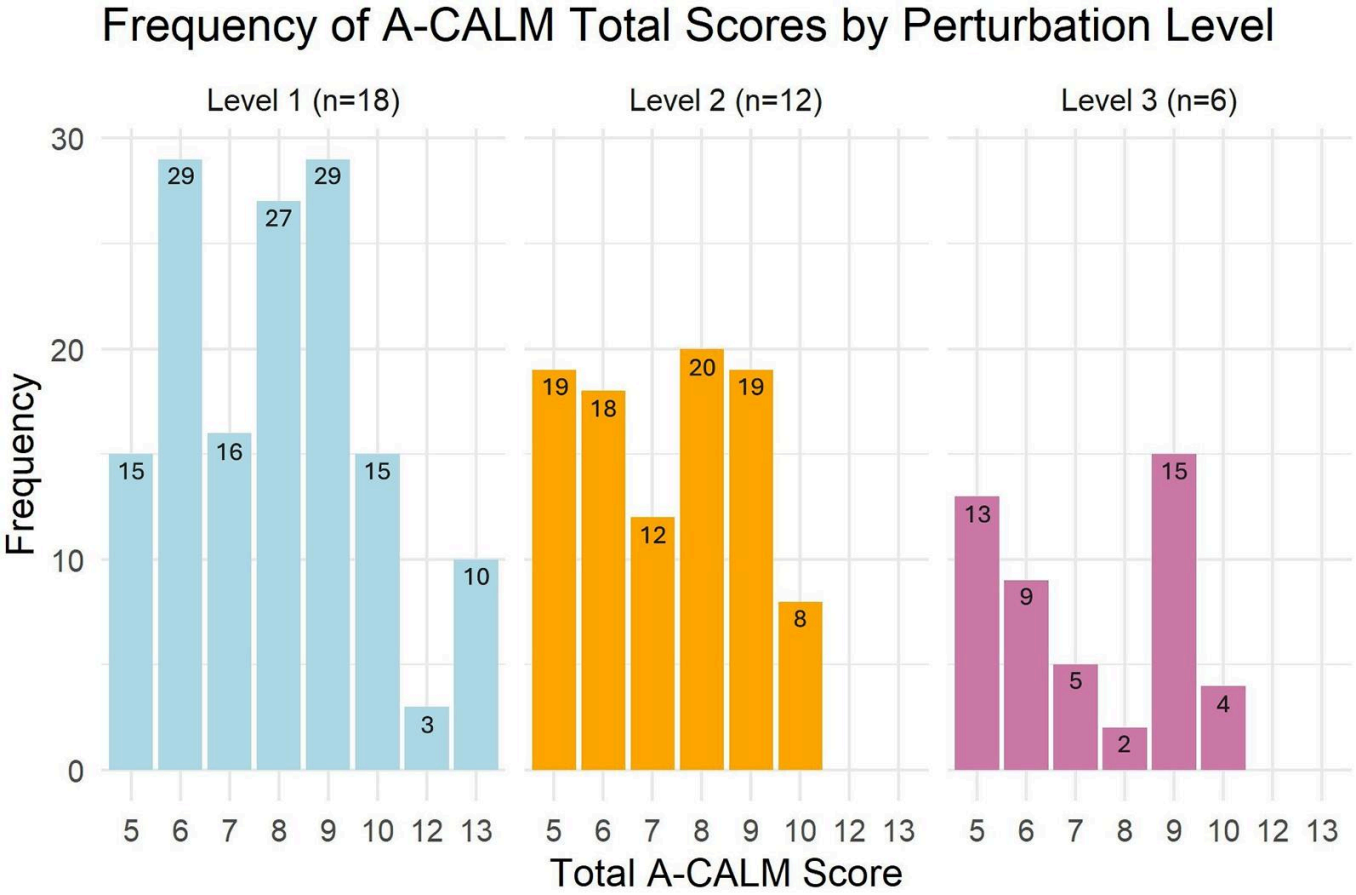
Frequency of the A-CALM total scores for different difficulty levels of the perturbations.

The distribution A-CALM total scores at each difficulty level is shown in Figure 2. At level 1, scores ranged from 5 to 13, with 6 and 9 occurring most frequently. At level 2, scores ranged from 5 to 10, with 8 and 5 most common. At level 3, scores again ranged from 5 to 10, with 9 and 5 most common.

Cumulative A-CALM scores showed median values of 15.5 (IQR = 13.75) for Level 1, 18.5 (IQR = 5.5) for Level 2, and 7 (IQR = 7.75) for Level 3. The wider IQR in Level 1 suggests greater variability in responses to lower-intensity perturbations, likely reflecting differences in baseline balance ability. The narrower IQR in Level 2 indicates more consistent performance under moderate intensity. Level 3 showed moderate variability, possibly reflecting the increased physical and cognitive demands of higher-intensity perturbations, where fewer participants-maintained performance. These differences in variability highlight the scale's potential sensitivity for detecting changes in reactive balance across varying task demands.

#### 3.2.3 Intra-rater reliability

The intra-rater reliability of the A-CALM total score was high, with a Cohen's Kappa of 0.86 (95% CI = 0.83–0.90) with significant values in all analyses (*p* < 0.001), indicating almost perfect agreement across time points (Table 5).

**Table 5:** Intra-rater reliability results.

A-CALM score	Cohen's Kappa	95% CI	*p*-value (*α* = 0.05)
Total score	0.86	(0.83–0.90)	*p* < 0.001
Arm score	0.85	(0.80–0.89)	*p* < 0.001
Leg score	0.80	(0.75–0.86)	*p* < 0.001

Cohen's Kappa values show the agreement between a single rater's first and second ratings for the A-CALM total score, arm score, and leg score. CI, confidence interval.

For the arm score, reliability was similarly strong (*K* = 0.85, 95% CI = 0.80–0.89). The leg score yielded a slightly lower value (*K* = 0.80, 95% CI = 0.75–0.86) but still demonstrated a high level of consistency in rating compensatory leg movements.

Comparisons between AP and ML perturbations revealed consistently higher reliability for AP directions. Arm scores in AP perturbations ranged from 0.78 to 0.83, all within the “substantial to almost perfect” range, with narrow confidence intervals (95% CI = 0.60–0.99) with significant values in all analyses (*p* < 0.001). ML arm scores were slightly lower (*K* = 0.61 to 0.71), falling in the “moderate to substantial” range, and showed wider confidence intervals (95% CI = 0.39–0.92). Leg scores followed a similar trend: AP values ranged from 0.74 to 0.84 (95% CI = 0.56–0.99), while ML values ranged from 0.47 to 0.77 (95% CI = 0.24–0.94). Some ML leg perturbations reached only moderate agreement, indicating greater variability in scoring. Overall, AP perturbations demonstrated stronger and more consistent reliability, suggesting they may offer a more stable basis for assessing reactive balance responses (see Supplementary Materials, Table S3).

The A-CALM cumulated score also demonstrated excellent intra-rater reliability across all three perturbation levels, with ICC values ≥0.93 (95% CI = 0.83–0.98) (see Supplementary Materials, Table S1).

In a secondary analysis, cumulative A-CALM scores at Level 1 demonstrated excellent reliability under both eight- and four-perturbation conditions. For eight perturbations, mean scores were 63.61 ± 9.71 at T1 and 67.11 ± 9.49 at T2, yielding an ICC(3,1) of 0.96 (95% CI: 0.89–0.98, *p* < 0.001). For four perturbations, mean scores were 31.44 ± 4.94 at T1 and 32.33 ± 5.14 at T2, with an ICC(3,1) of 0.96 (95% CI: 0.90–0.99, *p* < 0.001). These results indicate that four perturbations provide reliability comparable to eight, suggesting that fewer perturbations may be sufficient for a stable cumulative score (see Supplementary Materials, Table S4).

#### 3.2.4 Inter-rater reliability

The inter-rater reliability of the A-CALM total score was moderate, with a Fleiss' Kappa value of 0.41 (95% CI = 0.38–0.44) with significant values in all analyses (*p* < 0.001) across the three raters (Table 6). Agreement was higher for the arm score (*K* = 0.67, 95% CI = 0.62–0.72), while the leg score demonstrated moderate reliability (*K* = 0.48, 95% CI = 0.44–0.51).

**Table 6:** Inter-rater reliability results.

A-CALM score	Fleiss' Kappa	95% CI	*p*-value (*α* = 0.05)
Total score	0.41	(0.38–0.44)	*p* < 0.001
Arm score	0.67	(0.62–0.72)	*p* < 0.001
Leg score	0.48	(0.44–0.51)	*p* < 0.001

Fleiss' Kappa values show the agreement between the three raters for the A-CALM total, arm, and leg scores. CI, confidence interval.

Pairwise analyses showed substantial agreement between rater pairs for both the total score and arm scores, and moderate to substantial agreement for the leg score (Table 7).

**Table 7:** Inter-rater reliability results.

Score	Raters	Cohen's Kappa	95% CI	*p*-value (*α* = 0.05)
Total A-CALM Scale Score	R1-R2	0.74	(0.68–0.81)	*p* < 0.001
	R2-R3	0.72	(0.66–0.78)	*p* < 0.001
	R1-R3	0.69	(0.63–0.76)	*p* < 0.001
Total Arm Score	R1-R2	0.77	(0.71–0.83)	*p* < 0.001
	R2-R3	0.78	(0.73–0.84)	*p* < 0.001
	R1-R3	0.76	(0.71–0.82)	*p* < 0.001
Total Leg Score	R1-R2	0.66	(0.58–0.74)	*p* < 0.001
	R2-R3	0.61	(0.53–0.69)	*p* < 0.001
	R1-R3	0.58	(0.51–0.66)	*p* < 0.001

Cohen's Kappa values show the agreement between pairs of raters for the total A-CALM scale score, total leg score, total arm score. CI, confidence interval; R, rater.

For the cumulated A-CALM score, inter-rater reliability was good at Levels 1 and 2, with ICC(2,1) values ≥0.79 (95% CI = 0.34–0.94) with significant values in all analyses (*p* < 0.001). In contrast, Level 3 demonstrated poor reliability with an ICC(2,1) of 0.32 (95% CI = 0.02–0.63). These results suggest that raters reached higher consistency when assessing arm responses and lower perturbation levels, while agreement was weaker for leg responses and at the most challenging perturbations (see Supplementary Materials, Table S2).

## 4 Discussion

The A-CALM represents the first structured rater-based scale to systematically assess compensatory arm and leg movements during perturbations in treadmill walking. It was developed by adapting the original CALM scale, which was created for stance perturbations, to the walking context.

Using the A-CALM scale, three raters evaluated 288 perturbations and assigned scores accordingly. Reliability of individual perturbation ratings was examined with Cohen's Kappa, a statistic suited for categorical agreement and consistent with the approach used in the original CALM scale, allowing comparison with prior findings. For accumulated total scores, we applied the ICC, which better reflects overall consistency across continuous ratings.

Frequency analysis of A-CALM scores ranged 6–13, suggesting a potential ceiling effect at lower perturbation intensities but no evidence of a floor effect. At Level 1, the wide variability (IQR = 13.75) suggests that even mild perturbations elicited diverse responses, likely reflecting differences in baseline balance capacity or compensatory strategies. By contrast, Level 2 showed greater consistency (IQR = 5.5), suggesting that moderate perturbations present a more uniform challenge and may offer the most reliable conditions for assessing reactive balance across individuals. Level 3 produced lower median scores and moderate variability (IQR = 7.75), reflecting the higher physical and cognitive demands of intense perturbations, which prevented several participants from completing all perturbations.

Taken together, these findings suggest that the A-CALM scale is sensitive to performance differences across task difficulty levels. Level 2 appears most consistent for identifying balance capacity, while Levels 1 and 3 capture variability that may help distinguish individuals at different levels of fall risk.

The intra-rater reliability analysis shows that the A-CALM is a highly reliable tool for assessing compensatory arm and leg movements when applied by a single rater. This holds true for both the total score and the separate arm and leg subscores. Our findings suggest that, using the standardized A-CALM scale, a single rater was able to reproduce their initial assessments of compensatory movements two weeks later.

As expected, the A-CALM scale demonstrated slightly lower intra-rater reliability than the original CALM (10). Cohen's kappa values for the A-CALM were *K* = 0.86 (95% CI = 0.83–0.90) for the total score, *K* = 0.85 (95% CI = 0.80–0.89) for the arm score, and *K* = 0.80 (95% CI = 0.75–0.86) for the leg score, all statistically significant (*p* < 0.001). In comparison, the original CALM reported values of *K* ≥ 0.97 (95% CI = 0.91–1.00, *p* < 0.01). The modest reduction in reliability likely reflects the greater complexity of maintaining balance during dynamic walking perturbations compared to static stance tasks.

Nevertheless, the intra-rater reliability of the A-CALM remains high, supporting the success of its adaptation to treadmill-based perturbations. Its reliability is comparable to that of established tools, such as the Berg Balance Scale (*K* = 0.70–1.00, 95% CI = 0.98–0.99, *α* = 0.90) (28), and in many cases exceeds that of other measures, including the Clinical Gait and Balance Scale (*K* = 0.32–0.84, *p* < 0.01) (29). Despite the challenges of scoring reactive balance during perturbed walking—a task inherently variable and demanding—the A-CALM achieved levels of agreement on par with, or superior to, widely used clinical scales. This underscores its potential value as a research and clinical assessment tool.

The comparison between AP and ML perturbations revealed consistently higher reliability for AP perturbations. This is consistent with previous literature demonstrating that ML perturbations are more destabilizing and challenging to compensate for than AP perturbations (13). In the AP direction, individuals can use ankle strategies or take forward and backward steps within a relatively wider base of support, which likely contributes to more consistent scoring. In contrast, ML perturbations must be recovered within a narrower base of support and often require several rapid compensatory steps, increasing variability in scoring. These biomechanical constraints, coupled with the higher difficulty of ML recovery, likely explain the lower agreement observed. This finding aligns with evidence that ML instability is more strongly associated with fall risk than AP instability, particularly in older adults. Overall, the results highlight the clinical relevance of including directions in balance assessments, while recognizing that ML perturbations may serve as a more sensitive indicator of impaired balance control.

The inter-rater reliability analysis demonstrated that the A-CALM total score achieved moderate agreement across three raters (*K* = 0.41, 95% CI = 0.38–0.44) with significant values in all analyses (*p* < 0.001). The results suggest that while the scale is reliable when applied by a single rater, further refinement of item descriptions or enhanced rater training may be needed to improve consistency across multiple raters. This issue was most pronounced for leg movements, where the reliability was lower (*K* = 0.48, 95% CI = 0.44–0.51) compared to arm movements (*K* = 0.67, 95% CI = 0.62–0.72). One possible explanation is that video recordings from the frontal view made it difficult to identify the exact number of recovery steps, reducing consistency for leg scoring. In contrast, lateral arm movements were easier to observe from the same perspective, which may account for the higher reliability of arm assessments.

Although kappa values between 0.40 and 0.60 are typically classified as “moderate” agreement (22), some authors argue that values below 0.60 may offer limited confidence for clinical decision-making, particularly in high-stakes settings (30). Our values (0.41–0.48) fall at the lower end of this range, underscoring the need to improve inter-rater reliability—particularly for leg movement scoring. Future refinements could include clearer scoring criteria, enhanced rater training, and optimized camera positioning.

Pairwise comparisons between the individual raters showed a higher inter-rater reliability (*K* = 0.58–0.78, 95% CI = 0.51–0.84) compared to the three-rater analysis (*K* = 0.41–0.67, 95% CI = 0.38–0.72) with significant values in all analyses (*p* < 0.001). Consistent with previous findings, leg scores were slightly less reliable than arm scores. Notably, pairwise comparisons were similar across all rater combinations, which shows that reliability was not negatively affected by a single rater. Instead, the results suggest that reliability decreases as the number of raters increases.

The inter-reliability values of our study (*K* = 0.41–0.67, 95% CI = 0.38–0.72, *p* < 0.001) are somewhat lower than those of the original CALM (*K* = 0.46–0.87, 95% CI = 0.27–1.00, *p* < 0.01) (10). This suggests that adapting the scale from standing to walking conditions was accompanied by slight losses in reliability. Even so, the results show that the scale can be successfully applied to dynamic conditions, with overall reliability remaining comparable.

A previous study assessing balance recovery strategies through observer ratings reported higher inter-rater reliability than observed. Specifically, Cohen's Kappa values ranged from 0.96 to 0.98 (95% CI = 0.92–1.00, *p* < 0.001) for stepping strategies and from 0.91 to 0.99 (95% CI = 0.84–1.00, *p* < 0.001) for arm reactions (31). Unlike our protocol, that study evaluated participants who were standing quietly before perturbation, which created a clearer contrast between stillness and reactive movement. In walking, however, differentiating compensatory responses from normal gait movements is more challenging, making observation inherently more complex. Moreover, the previous study applied a coarser rating scale (e.g., presence vs. absence of arm movement), where the A-CALM scale uses a finer 5-point Likert scale for arm use. Although this resolution provides richer detail on reactive balance strategies, it may also reduce inter-rater reliability, especially when multiple raters are involved.

Most ICC values fell within the good-to-excellent range, indicating that the A-CALM scale can reliably assess reactive balance during an 8-perturbation walking block. These values were somewhat lower than the strong inter-rater ICCs reported for global balance scales such as the Mini-BESTest (ICC = 0.95) (32). In contrast, intra-rater ICCs of the A-CALM (ICC = 0.93–0.96) exceeded those of the Berg Balance Scale (ICC = 0.82) or Dynamic Gait Index (ICC = 0.87) (33).

Most ICCs for the A-CALM also exceeded those reported for other reactive balance assessments. For example, the Reactive Balance Test in patients with chronic ankle instability demonstrated ICCs ranging from 0.69 to 0.87 (95% CI = 0.42–0.94) (34). The narrow confidence intervals of intra-rater reliability indicate that the A-CALM is robust for tracking individual changes over time. In contrast, wider inter-rater confidence intervals suggest that consistent scoring by the same rater or additional rater training may be needed to enhance broader clinical applicability.

For the most challenging perturbations (Level 3), intra-rater reliability for the cumulative A-CALM score was low (ICC = 0.32, 95% CI = 0.02–0.63) with significant values in all analyses (*p* < 0.001) (see Supplementary Materials, Table S2). However, this analysis was based on a small sample (*n* = 6), so these results should be interpreted cautiously and validated in larger cohorts.

The similarity in score distributions between levels 2 and 3 may reflect participant characteristics rather than scale limitations. Only the most physically fit participants (*n* = 6) reached Level 3, and their higher baseline postural stability may have allowed performance comparable to participants at Level 2 (*n* = 12), despite the increased task difficulty.

Ceiling effects at lower difficulty levels likely reflect the limited challenge posed by small perturbations for our healthy, community-dwelling older adult sample, who generally maintained balance with minimal compensatory movements. This restricted variability indicates that these levels may have limited discriminatory value for high-functioning individuals but remain important for populations with greater balance impairments, where performance may differ more substantially.

An important consideration when interpreting A-CALM scale scores is that the scale evaluates balance recovery responses, not fall risk directly. Lower scores reflect greater reliance on compensatory strategies such as stepping or large limb movements, but these strategies may still be highly effective in preventing a fall. Thus, the A-CALM scale is particularly valuable for distinguishing how individuals recover from perturbations, rather than predicting who will fall. For example, two individuals may both avoid falling, yet one does so with minimal adjustment while another requires multiple steps or marked arm movements. This distinction reflects differences in postural robustness that are clinically and functionally meaningful, even if both outcomes appear successful. In this way, the A-CALM scale complements existing fall-risk tools by providing a more nuanced assessment of reactive balance control under unpredictable, high-challenge conditions.

We selected a single frontal-plane camera to capture lateral compensatory movements while maintaining a low-cost, simple, and clinically transferable setup. Although a sagittal view could improve detail for AP responses, the added complexity may reduce feasibility in routine clinical use.

Widely used clinical tools such as the Functional Gait Assessment (FGA) (35) and the Mini-BESTest (36) provide comprehensive evaluations of gait, dynamic stability, and multiple balance domains. However, they do not explicitly quantify compensatory arm and leg movements during reactive balance tasks. The A-CALM scale complements these measures by targeting a specific and clinically relevant aspect of postural control—the magnitude and quality of limb responses following unexpected perturbations. This targeted scoring can uncover deficits in reactive control that remain undetected by broader functional assessments, thereby enabling more precise evaluation and intervention planning.

Our findings, consistent with the original CALM scale (10), indicate that a single perturbation is insufficient to reliably assess reactive balance. To address this, the A-CALM scale includes eight perturbations per level, capturing a wider range of responses and reducing variability from chance or isolated performance anomalies. While our main analysis focused on the complete set of eight perturbations, it is also important to identify the minimum number needed to achieve acceptable reliability. For this purpose, we conducted additional analyses using ICC calculations on cumulative scores derived from four perturbations (e.g., one in each direction) to evaluate whether a reduced set can still provide robust and reliable assessments.

Our subanalysis indicates that the A-CALM scale demonstrates excellent intra-rater reliability at Level 1, whether cumulative scores are based on eight or reduced to four. The identical ICC values (ICC = 0.96) suggest that reducing the number of perturbations does not meaningfully affect score stability. This finding is practically important: using only four perturbations reduces testing time and participant burden while preserving measurement precision. The high reliability of the four-perturbation condition further supports the idea that a smaller subset can adequately capture compensatory movement responses, making the protocol more feasible for clinical and research use. Based on our estimates of a two-min rating time per perturbation, the time required to rate the A-CALM with four perturbations is approximately 8 min. These estimates are clinically relevant because they provide a realistic indication of the time and training investment required for reliable use of the A-CALM scale in practice. With a manageable scoring time per participant, this scale has the potential to be feasibly implemented in both research and clinical settings to evaluate reactive balance responses.

Our results are consistent with current clinical practice guidelines and implementation frameworks (37) that emphasize the need for standardized, reliable reactive balance assessments in populations at risk of falls. Reactive postural control is a key modifiable factor in fall prevention, yet remains underassessed in rehabilitation practice. By showing that the A-CALM scale can be applied reliably in older adults, our study provides an accessible method that helps overcome common implementation barriers such as limited time, lack of appropriate tools, and safety concerns during high-challenge testing. Integrating the A-CALM scale into practice could complement existing anticipatory balance assessments and provide more tailored insights for fall prevention interventions.

Unlike laboratory-based reactive balance assessments, which often require specialized equipment (e.g., motion capture systems or force plates) and trained technical staff, the A-CALM scale can be administered with standard video recording equipment. This approach substantially lowers equipment costs, reduces setup time, and eliminates the need for specialized operators. As a result, the A-CALM scale represents a more accessible and cost-effective option for both clinical and research settings, particularly in contexts with limited resources.

### 4.1 Limitations and future research

This study has several limitations that should be considered. First, we relied on a single perturbation modality using the BalanceTutor® treadmill (MediTouch Ltd., Israel). It is unclear whether the findings can be generalized to other perturbation systems (e.g., split belt treadmills) or to alternative methods such as manual pushes/pulls, movable obstacles, or slip/trip devices. Different perturbation mechanics—such as underfoot changes in ground reaction forces vs. external applied disturbances—may elicit distinct compensatory strategies, potentially affecting A-CALM score distributions. Dedicated studies are needed to determine whether the scoring principles remain robust across diverse perturbation types and environments.

Second, the operational definition of a compensatory step was intentionally simple, defined as any deviation from normal gait involving an adjustment in foot placement to preserve balance. While this enhances clinical usability, it may not always allow clear distinction between compensatory and regular steps, particularly in the AP direction where changes can be subtle. Refining this definition could improve scoring precision and reliability in future applications.

A further limitation related to the smaller number of participants who were able to complete Levels 2 and 3, which represent the highest balance challenges within the A-CALM scale. As a result, the reliability estimates for these levels are less certain, and additional studies are needed to confirm the scale's performance under more extreme balance conditions.

Another important consideration is that repeated assessments in this study were performed by a single rater. Although this allowed us to establish strong intra-rater reliability, it limits conclusions about generalizability across different raters. Including multiple raters in future studies will be essential to better capture inter-rater variability and strengthen the overall evidence base for the A-CALM scale's reliability.

Finally, while this study demonstrated reliability comparable to that of the original CALM scale, we did not investigate validity. The original CALM demonstrated significant correlations with kinematic measurement parameters (*r* = −0.48 to −0.91, *p* < 0.01), supporting its criterion validity against a gold-standard reference. Because the present study took place in a clinical environment, kinematic analysis was not available. Future research should therefore establish the validity of the A-CALM scale by comparing its ratings with gold-standard kinematic data collected during perturbation treadmill walking. Quantifying biomechanical parameters such as recovery step count and arm swing amplitude after perturbations and correlating them with A-CALM ratings will be critical to confirm whether the scale captures the underlying mechanisms of reactive balance recovery.

### 4.2 Conclusion

Our results demonstrate that compensatory leg and arm movements can be reliably assessed using the A-CALM scale. At the same time, the findings highlight areas for refinement, particularly in the scoring of leg movements, where subjectivity may limit consistency. Future efforts should focus on improving scoring criteria to enhance clarity and inter-rater agreement.

Further research is also needed to establish the validity of the A-CALM, including its predictive value for clinically relevant outcomes such as falls and incident disability. Demonstrating such associations would strengthen its utility and make the scale more appealing to clinicians in the fall-prevention practice.

In summary, the A-CALM scale showed strong intra-rater reliability and moderate to substantial inter-rater reliability, supporting its potential for consistent use across raters and over time. By addressing current limitations and pursuing systematic validation, the A-CALM can evolve into a robust, clinically practical tool for evaluating reactive balance and compensatory strategies during walking. Ultimately, the scale offers a promising pathway to enhance assessment practices, inform rehabilitation, and contribute to more effective fall-prevention strategies for individuals at risk of balance impairments.

## Data Availability

The raw data supporting the conclusions of this article will be made available by the authors, without undue reservation.
